# Editorial: Advances in the Involvement of Human Papilloma Virus in Head and Neck Squamous Cell Carcinoma

**DOI:** 10.3389/fonc.2022.795280

**Published:** 2022-03-03

**Authors:** Jerome R. Lechien, Francois Mouawad, Stéphane Hans, Sven Saussez

**Affiliations:** ^1^ Department of Human Anatomy and Experimental Oncology, Faculty of Medicine, UMONS Research Institute for Health Sciences and Technology, University of Mons (UMons), Mons, Belgium; ^2^ Department of Otolaryngology-Head and Neck Surgery, Foch Hospital, School of Medicine, University Paris Saclay, Paris, France; ^3^ Department of Otolaryngology, Elsan Hospital, Paris, France; ^4^ Department of Otolaryngology-Head and Neck Surgery, CHRU de Lille, Hopital Claude Huriez, Lille, France; ^5^ Department of Otolaryngology-Head and Neck Surgery, Centre Hospitalier Universitaire (CHU) Saint-Pierre, Brussels, Belgium

**Keywords:** cancer, head neck, HPV, human papillomavirus, carcinoma, otolaryngology, head neck, surgery

## Background

Head and neck squamous cell carcinomas (HNSCC) are the sixth most common cancers in males and the eighth most common in females worldwide, accounting for over 890,000 new cases in 2017 (1, 2). HNSCCs represent 5.3% of all cancers. From a mortality standpoint, they involve more than 500,000 deaths worldwide, representing 5.3% of all cancer deaths (2). Irrespective to the world region, the most prevalent HNSCCs in 2017 were lip and oral cavity (390,000 new cases), laryngeal (211,000 new cases), pharyngeal (179,000 new cases) and nasopharyngeal (110,000 new cases) carcinomas (2). The main risk factors for HNSCCs are alcohol and tobacco consumption and persistent infection with human papillomavirus (HPV) that is associated with the rising incidence of oropharyngeal squamous cell carcinoma (OSCC) in the United States and Europe (3, 4). The incidence of HPV-induced HNSCC depends on the world region and the method used to assess the presence of HPV (5). The incidence of HPV-induced HNSCC has increased since the 80s (5).

HPV infection is predominantly attributable to subtypes HPV-16 and HPV-18 even if there is geographical heterogeneity between continents (3, 6, 7). Young, nondrinking and nonsmoking individuals with HPV-induced OSCC often have advanced carcinoma, but they have a better prognosis compared with patients with tobacco- and alcohol-induced OSCC (6, 7). The mechanisms underlying the better prognosis of patients with HPV-induced HNSCC remain poorly understood and may involve interactions between HPV antigens and the host-immune system, leading to an improved responses of immune cells against tumor (8). In that way, an increasing number of studies reported significant differences in the composition of tumor-infiltrating immune cells in HPV-positive and HPV-negative HNSCC (8). Most authors recognized that the overexpression of some immune cells may be considered as a significant prognostic factor for HNSCC patients (8). In this Research Topic, several research groups published clinical and experimental studies about the involvement of HPV in the development of OSCC and non-OSCC.2

## Immunological Features of HPV-Induced Carcinoma

The better survival outcomes of patients with HPV-positive OSCC may be explained by immunological differences between HPV-positive and HPV-negative HNSCCs. The HPV infection is associated with an increased CD8+ T tumor cell infiltration and PD-1 expression by CD8+ T cells in both OSCC and non-OSCC, which is associated with better overall survival (OS). The interaction between the tumor environment and CD8+ T cells is mediated through many cytokines, including IFN-g and IL-2, -4, -8, -12 and -17 (9, 10). Recent studies supported interactions between CD8+ and CD4+ T cells in HPV-positive HNSCC (8). Precisely, in tumor environment, CD4+ T cells may be converted into Th17 cells, which could potentiate the cytotoxic effects of CD8+ T cells against HPV-induced tumor cells (8). M1 and M2 macrophages are additional immune cells that may have an important role in HPV-induced carcinogenesis (11). M2 macrophages are involved in the enhancement of immunosuppression through the stimulation of regulatory T cells (Tregs) that induces a favorable environment for tumor growth through the secretion of TGF-b, TNF-a, and IL-10 (8). In HPV-negative HNSCC, an increased proportion of M2 cells is an important factor underlying the improvement of OS (8). The involvement of Foxp3 Tregs in HPV-positive and HPV-negative HNSCC remains uncertain. Indeed, several researches showed that an increase of Tregs in HPV-positive HNSCC environment was associated with improved OS (8, 12), while others reported similar findings in HNSCC but no influence of the HPV status (8, 13). Foxp3 Tregs may inhibit the protumoral effects of inflammatory immune cells, which is a favorable prognostic marker at some tumor sites, whereas in other tumor sites, Treg infiltration may be associated with poor survival outcomes regarding their conventional regulatory function (13). The activation of Tregs may be associated with myeloid derived suppressor cells infiltration, these cells being associated with tumor progression and PD-L1 expression (14).

## Limitations and Heterogeneity Studies

The findings of studies investigating the immune cell features of HPV-induced HNSCCs have to be considered with cautious because many limitations. The method used to determine the HPV status may significantly vary from one to another study. In many centers, the detection of HPV infection is made with p16 immunostaining. Some authors used p16 immunostaining to compare data from HPV-positive and HPV-negative HNSCCs, while others used polymerase chain reaction (PCR) or other direct methods of DNA identification (15). In fact, some tumors can be HPV-positive/p16- or HPV-negative/p16+, which can be related to the lack of specificity of p16 in identifying HPV infection. As demonstrated in two studies (15, 16), the use of p16 immunostaining *versus* DNA detection may lead to substantial differences in the study results, which may bias the comparison between studies.

From an epidemiological standpoint, the analysis of study results (e.g. OS, disease-free survival) has to consider inclusion criteria. For example, the survival analysis according to the HPV stratus has to consider the ethnicity, tumor site, tobacco and alcohol histories, and the types of treatment (e.g. surgery, chemoradiation, radiotherapy or immunotherapy) (15); all of these points being known to influence the immune cell infiltrate.

## Perspectives and Conclusion

Epidemiological, clinical, and immunological outcomes of HPV-induced HNSCCs are all points investigated in studies of this Research Topic. Precisely, the following outcomes were studied in the present Research Topic: prevalence data of HPV-induced HNSCC in Syria (16), importance of extranodal extension HPV-positive OSCC (Gupta et al.), tumor microenvironment and immunotherapy in OSCC (Beltz et al.), survival outcomes according to lymph node invasion in oral SCC (Welters et al.), nonsmoking and nondrinking outcomes in HNSCC (Li et al.; Yang et al.), the impact of tonsillectomy in the management of unknown primary HNSCC regarding p16 status (Mulder et al.), and recurrent respiratory papillomatosis risk factors (Podeur et al.). Many grey areas have to be explored in the next few years to better understand the prognosis differences between HPV-positive and HPV-negative HNSCC patients, as well as the influence of some conditions on both immunological and survival features. A promising topic is the study of the influence of gut, laryngopharyngeal and oral microbiota on the immune regulation in tumor environment and its interaction with HPV infection.

## Author Contributions

All authors listed have made a substantial, direct, and intellectual contribution to the work and approved it for publication.

## Conflict of Interest

The authors declare that the research was conducted in the absence of any commercial or financial relationships that could be construed as a potential conflict of interest.

## Publisher’s Note

All claims expressed in this article are solely those of the authors and do not necessarily represent those of their affiliated organizations, or those of the publisher, the editors and the reviewers. Any product that may be evaluated in this article, or claim that may be made by its manufacturer, is not guaranteed or endorsed by the publisher.
